# *catena*-Poly[[(1,12,15,26-tetra­aza-5,8,19,22-tetra­oxa-3,4:9,10:17,18:23,24-tetra­benzocyclo­octa­cosane-κ^4^ *N* ^1^,*N* ^12^,*N* ^15^,*N* ^26^)nickel(II)]-μ-terephthalato-κ^2^ *O* ^1^:*O* ^4^]

**DOI:** 10.1107/S160053680803314X

**Published:** 2008-10-18

**Authors:** Shuang-Ming Meng, Yue-Qin Fan, Yong Guo

**Affiliations:** a College of Chemistry and Chemical Engineering, Shanxi Datong University, Datong 037009, People’s Republic of China

## Abstract

In the title compound, [Ni(C_8_H_4_O_4_)(C_36_H_44_N_4_O_4_)]_*n*_, the Ni^II^ atom is coordinated in a distorted octa­hedral geometry by the four N atoms of the 1,12,15,26-tetra­aza-5,8,19,22-tetra-oxa-3,4:9,10:17,18:23,24-tetra­benzocyclo­octa­cosane ligand and two O atoms from the terephthalate dianions. The Ni^II^ atoms, which lie on inversion centres, are linked *via* terephthalate ligands to form a chain structure along [101]. The structure is stabilized by three intra­molecular and one inter­molecular N—H⋯O hydrogen bonds.

## Related literature

For general background, see: Choi & Suh (1999 ▶); Massoud *et al.* (2006 ▶); Ray *et al.* (2006 ▶). For a related structure, see: Jiang *et al.* (2005 ▶).
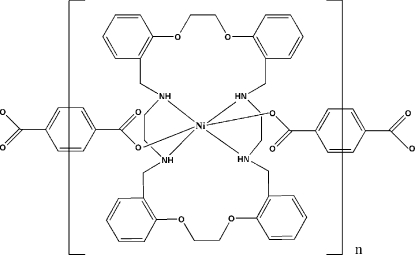


## Experimental

### 

#### Crystal data

[Ni(C_8_H_4_O_4_)(C_36_H_44_N_4_O_4_)]
*M* *_r_* = 819.57
Monoclinic, 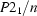

*a* = 11.407 (3) Å
*b* = 16.575 (3) Å
*c* = 21.675 (6) Å
β = 101.758 (10)°
*V* = 4012.2 (17) Å^3^
*Z* = 4
Mo *K*α radiation
μ = 0.54 mm^−1^
*T* = 293 (2) K
0.35 × 0.28 × 0.21 mm

#### Data collection

Rigaku R-AXIS RAPID diffractometer
Absorption correction: multi-scan (*ABSCOR*; Higashi, 1995 ▶) *T*_min_ = 0.839, *T*_max_ = 0.910
37289 measured reflections
9133 independent reflections
5535 reflections with *I* > 2σ(*I*)
*R*_int_ = 0.095

#### Refinement

*R*[*F* ^2^ > 2σ(*F* ^2^)] = 0.063
*wR*(*F* ^2^) = 0.155
*S* = 1.05
9133 reflections
514 parameters
4 restraints
H atoms treated by a mixture of independent and constrained refinement
Δρ_max_ = 0.92 e Å^−3^
Δρ_min_ = −0.79 e Å^−3^

### 

Data collection: *PROCESS-AUTO* (Rigaku, 1998 ▶); cell refinement: *PROCESS-AUTO*; data reduction: *PROCESS-AUTO*; program(s) used to solve structure: *SHELXS97* (Sheldrick, 2008 ▶); program(s) used to refine structure: *SHELXL97* (Sheldrick, 2008 ▶); molecular graphics: *SHELXTL-Plus* (Sheldrick, 2008 ▶); software used to prepare material for publication: *SHELXL97*.

## Supplementary Material

Crystal structure: contains datablocks global, I. DOI: 10.1107/S160053680803314X/pv2108sup1.cif

Structure factors: contains datablocks I. DOI: 10.1107/S160053680803314X/pv2108Isup2.hkl

Additional supplementary materials:  crystallographic information; 3D view; checkCIF report

## Figures and Tables

**Table 1:** Hydrogen-bond geometry (Å, °)

*D*—H⋯*A*	*D*—H	H⋯*A*	*D*⋯*A*	*D*—H⋯*A*
N1—H1N⋯O1	0.83 (2)	2.58 (4)	3.101 (4)	122 (3)
N2—H2N⋯O8^i^	0.81 (2)	2.26 (2)	3.003 (4)	152 (4)
N3—H3N⋯O3	0.84 (2)	2.47 (3)	3.044 (4)	127 (3)
N4—H4N⋯O5	0.83 (2)	2.14 (3)	2.862 (4)	144 (4)

Symmetry codes: (i) 
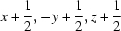
; (ii) 
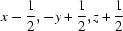
; (iii) 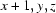.

**Fig. 1.**
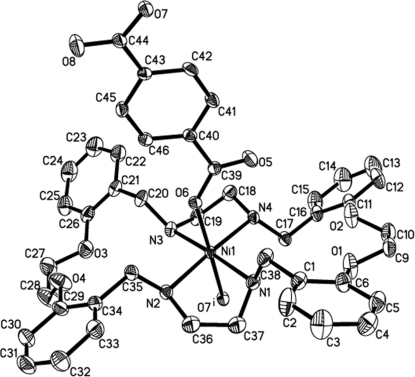
Molecular structure of the title compound. Displacement ellipsoids are drawn at the 30% probability level and H atoms have been omitted for clarity. [Symmetry code: (i) x + 1/2, 1/2 - y, 1/2 + z]

**Fig. 2.**
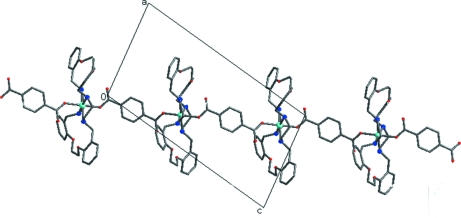
View of the one-dimensional polymeric chain of the title compound in the unit cell; H atoms have been omitted for clarity.

## supplementary crystallographic information

### Comment

In recent years, intense research activity has been directed toward the assembly of carboxylato-bridged macrocyclic polymers due to their intriguing multidimensional networks (Choi & Suh, 1999; Massoud *et al.* 2006; Ray *et al.* 2006). As an extension of the research on the macrocyclic complexes, we have prepared the title compound, (I). In this paper the crystal structure of (I) is reportd.

In the title compound, the Ni^II^ atom, which lies on an inversion centre, displays a distorted octahedral coordination geometry provided by four nitrogen atoms from the ligand, 3,4:9,10:17,18:23,24-tetrabenzo- 1,12,15,26-tetraaza-5,8,19,22-tetraoxacyclooctacosane (*L*) and two oxygen atoms from two distinct terephthalate (tp) dianion ligands (Fig. 1). The bond distances and angles show normal values (Jiang *et al.* 2005). The Ni^II^ atoms are linked *via * tp ligands to form a one-dimensional chain structure (Fig. 2). The constituent of the title compound are linked through hydrogen bonds to form a complicated three-dimensional network (Table 1); the N and C atoms play a role as donors, while carboxylate-O atoms function as acceptors in these hydrogen bonds.

### Experimental

A mixture of NiCO_3_ (0.119 mg, 1 mmol), terephthalic acid (0.162 mg, 1 mmol) and 3,4:9,10:17,18:23,24-tetrabenzo-1,12,15,26-tetraaza-5,8,19,22 -tetraoxacyclooctacosane (0.596 mg, 1 mmol) in EtOH (10 ml) was placed in a Teflon reactor and heated at 393 K for 3 days, and then it was gradually cooled to room temperature at a rate of 10 K.h^-1^. Green crystals were obtained.

### Refinement

All H atoms bound to C atoms were positioned geometrically and refined as riding, with C—H = 0.93 Å (CH) and 0.97 Å (CH_2_) and *U*_iso_(H) = 1.2*U*_eq_(C). The H atoms bound to N atoms were located in a difference Fourier map and refined with *U*_iso_(H) = 1.2*U*_eq_(N).

### Crystal data

**Table:**

[Ni(C_8_H_4_O_4_)(C_36_H_44_N_4_O_4_)]	*F*(000) = 1728
*M**_r_* = 819.57	*D*_x_ = 1.357 Mg m^−^^3^
Monoclinic, *P*2_1_/*n*	Mo *K*α radiation, λ = 0.71073 Å
Hall symbol: -P 2yn	Cell parameters from 9133 reflections
*a* = 11.407 (3) Å	θ = 3.1–27.5°
*b* = 16.575 (3) Å	µ = 0.54 mm^−^^1^
*c* = 21.675 (6) Å	*T* = 293 K
β = 101.758 (10)°	Block, green
*V* = 4012.2 (17) Å^3^	0.35 × 0.28 × 0.21 mm
*Z* = 4	

### Data collection

**Table:**

Rigaku R-AXIS RAPID diffractometer	9133 independent reflections
Radiation source: fine-focus sealed tube	5535 reflections with *I* > 2σ(*I*)
graphite	*R*_int_ = 0.095
Detector resolution: 10.0 pixels mm^-1^	θ_max_ = 27.5°, θ_min_ = 3.1°
ω scans	*h* = −14→14
Absorption correction: multi-scan (ABSCOR; Higashi, 1995)	*k* = −21→21
*T*_min_ = 0.839, *T*_max_ = 0.910	*l* = −27→28
37289 measured reflections	

### Refinement

**Table:**

Refinement on *F*^2^	Primary atom site location: structure-invariant direct methods
Least-squares matrix: full	Secondary atom site location: difference Fourier map
*R*[*F*^2^ > 2σ(*F*^2^)] = 0.063	Hydrogen site location: inferred from neighbouring sites
*wR*(*F*^2^) = 0.155	H atoms treated by a mixture of independent and constrained refinement
*S* = 1.05	*w* = 1/[σ^2^(*F*_o_^2^) + (0.0632*P*)^2^ + 2.1456*P*] where *P* = (*F*_o_^2^ + 2*F*_c_^2^)/3
9133 reflections	(Δ/σ)_max_ = 0.001
514 parameters	Δρ_max_ = 0.92 e Å^−^^3^
4 restraints	Δρ_min_ = −0.79 e Å^−^^3^

### Special details

**Table:**

Geometry. All e.s.d.'s (except the e.s.d. in the dihedral angle between two l.s. planes) are estimated using the full covariance matrix. The cell e.s.d.'s are taken into account individually in the estimation of e.s.d.'s in distances, angles and torsion angles; correlations between e.s.d.'s in cell parameters are only used when they are defined by crystal symmetry. An approximate (isotropic) treatment of cell e.s.d.'s is used for estimating e.s.d.'s involving l.s. planes.
Refinement. Refinement of *F*^2^ against ALL reflections. The weighted *R*-factor *wR* and goodness of fit *S* are based on *F*^2^, conventional *R*-factors *R* are based on *F*, with *F* set to zero for negative *F*^2^. The threshold expression of *F*^2^ > σ(*F*^2^) is used only for calculating *R*-factors(gt) *etc*. and is not relevant to the choice of reflections for refinement. *R*-factors based on *F*^2^ are statistically about twice as large as those based on *F*, and *R*- factors based on ALL data will be even larger.

### Fractional atomic coordinates and isotropic or equivalent isotropic displacement parameters (Å^2^)

**Table:**

	*x*	*y*	*z*	*U*_iso_*/*U*_eq_	
Ni1	0.76937 (4)	0.21301 (2)	0.898586 (19)	0.02536 (13)	
C1	0.5251 (3)	0.0013 (2)	0.84876 (18)	0.0422 (9)	
C2	0.5463 (4)	−0.0627 (3)	0.8114 (2)	0.0660 (14)	
H2	0.6008	−0.0565	0.7852	0.079*	
C3	0.4876 (5)	−0.1363 (3)	0.8124 (3)	0.0747 (16)	
H3	0.5032	−0.1786	0.7871	0.090*	
C4	0.4075 (4)	−0.1459 (3)	0.8502 (2)	0.0628 (13)	
H4	0.3674	−0.1947	0.8505	0.075*	
C5	0.3859 (4)	−0.0837 (3)	0.8880 (2)	0.0573 (12)	
H5	0.3307	−0.0902	0.9138	0.069*	
C6	0.4457 (4)	−0.0118 (2)	0.8878 (2)	0.0479 (10)	
C9	0.3299 (4)	0.0644 (3)	0.9479 (2)	0.0519 (11)	
H9A	0.3091	0.0169	0.9696	0.062*	
H9B	0.2668	0.0734	0.9112	0.062*	
C10	0.3383 (4)	0.1346 (3)	0.9901 (2)	0.0623 (13)	
H10A	0.2583	0.1526	0.9920	0.075*	
H10B	0.3779	0.1185	1.0322	0.075*	
C11	0.4113 (3)	0.2674 (2)	1.00719 (17)	0.0414 (9)	
C12	0.3125 (4)	0.3033 (3)	1.0236 (2)	0.0643 (13)	
H12	0.2367	0.2809	1.0106	0.077*	
C13	0.3278 (5)	0.3727 (4)	1.0595 (3)	0.0872 (18)	
H13	0.2621	0.3977	1.0706	0.105*	
C14	0.4397 (6)	0.4046 (4)	1.0786 (3)	0.0852 (18)	
H14	0.4500	0.4506	1.1037	0.102*	
C15	0.5377 (5)	0.3695 (3)	1.0612 (2)	0.0641 (13)	
H15	0.6131	0.3923	1.0742	0.077*	
C16	0.5245 (3)	0.3001 (2)	1.02442 (17)	0.0395 (9)	
C17	0.6303 (3)	0.2610 (2)	1.00410 (16)	0.0389 (9)	
H17A	0.7025	0.2758	1.0340	0.047*	
H17B	0.6216	0.2029	1.0062	0.047*	
C18	0.6834 (4)	0.3678 (2)	0.93574 (19)	0.0457 (10)	
H18A	0.6499	0.4007	0.9649	0.055*	
H18B	0.6541	0.3882	0.8935	0.055*	
C19	0.8183 (4)	0.3724 (2)	0.95182 (19)	0.0476 (10)	
H19A	0.8440	0.4281	0.9509	0.057*	
H19B	0.8479	0.3512	0.9938	0.057*	
C20	0.8686 (4)	0.3707 (2)	0.84692 (18)	0.0435 (9)	
H20A	0.7875	0.3871	0.8282	0.052*	
H20B	0.8962	0.3354	0.8171	0.052*	
C21	0.9472 (4)	0.4448 (2)	0.85675 (18)	0.0429 (9)	
C22	0.8995 (5)	0.5222 (2)	0.8504 (2)	0.0600 (12)	
H22	0.8169	0.5296	0.8406	0.072*	
C23	0.9760 (6)	0.5892 (3)	0.8586 (3)	0.0780 (17)	
H23	0.9442	0.6410	0.8549	0.094*	
C24	1.0953 (6)	0.5787 (3)	0.8720 (3)	0.0761 (16)	
H24	1.1452	0.6236	0.8768	0.091*	
C25	1.1447 (5)	0.5027 (3)	0.8786 (2)	0.0646 (13)	
H25	1.2274	0.4961	0.8883	0.077*	
C26	1.0705 (4)	0.4364 (2)	0.8707 (2)	0.0487 (10)	
C27	1.2162 (4)	0.3402 (3)	0.8544 (3)	0.0679 (14)	
H27A	1.2857	0.3572	0.8853	0.081*	
H27B	1.2160	0.3694	0.8156	0.081*	
C28	1.2233 (4)	0.2533 (3)	0.8431 (3)	0.0801 (17)	
H28A	1.2944	0.2420	0.8265	0.096*	
H28B	1.2305	0.2247	0.8827	0.096*	
C29	1.1211 (4)	0.1467 (2)	0.78328 (19)	0.0444 (9)	
C30	1.2200 (4)	0.1073 (3)	0.7688 (2)	0.0617 (13)	
H30	1.2932	0.1336	0.7733	0.074*	
C31	1.2076 (5)	0.0288 (3)	0.7478 (2)	0.0674 (14)	
H31	1.2738	0.0016	0.7393	0.081*	
C32	1.0996 (5)	−0.0101 (3)	0.7394 (2)	0.0665 (13)	
H32	1.0920	−0.0628	0.7243	0.080*	
C33	1.0018 (4)	0.0298 (3)	0.7534 (2)	0.0561 (11)	
H33	0.9281	0.0038	0.7470	0.067*	
C34	1.0114 (4)	0.1088 (2)	0.77716 (18)	0.0423 (9)	
C35	0.9067 (3)	0.1515 (2)	0.79519 (17)	0.0388 (9)	
H35A	0.9145	0.2089	0.7883	0.047*	
H35B	0.8338	0.1334	0.7674	0.047*	
C36	0.8639 (4)	0.0525 (3)	0.8715 (3)	0.0643 (9)	
H36A	0.8305	0.0284	0.8310	0.077*	
H36B	0.9374	0.0239	0.8890	0.077*	
C37	0.7792 (4)	0.0404 (3)	0.9134 (3)	0.0643 (9)	
H37A	0.7457	−0.0135	0.9075	0.077*	
H37B	0.8211	0.0453	0.9569	0.077*	
C38	0.5845 (4)	0.0823 (2)	0.84676 (19)	0.0474 (10)	
H38A	0.6153	0.0855	0.8083	0.057*	
H38B	0.5243	0.1240	0.8449	0.057*	
C39	0.5795 (3)	0.2704 (2)	0.78663 (15)	0.0322 (8)	
C40	0.5548 (3)	0.2906 (2)	0.71679 (15)	0.0295 (7)	
C41	0.4375 (3)	0.2997 (2)	0.68456 (16)	0.0364 (8)	
H41	0.3755	0.2989	0.7066	0.044*	
C42	0.4119 (3)	0.3100 (2)	0.61992 (16)	0.0350 (8)	
H42	0.3326	0.3154	0.5990	0.042*	
C43	0.5016 (3)	0.3125 (2)	0.58600 (15)	0.0293 (7)	
C44	0.4709 (3)	0.3139 (2)	0.51426 (16)	0.0335 (8)	
C45	0.6199 (3)	0.3074 (2)	0.61860 (16)	0.0371 (9)	
H45	0.6820	0.3117	0.5969	0.044*	
C46	0.6456 (3)	0.2961 (2)	0.68337 (16)	0.0360 (8)	
H46	0.7249	0.2921	0.7046	0.043*	
O1	0.4368 (3)	0.05093 (19)	0.92886 (16)	0.0669 (7)	
O2	0.4008 (3)	0.19793 (19)	0.97062 (16)	0.0669 (7)	
O3	1.1119 (3)	0.35817 (18)	0.87622 (17)	0.0655 (9)	
O4	1.1251 (3)	0.22632 (19)	0.80180 (18)	0.0706 (10)	
O5	0.4986 (2)	0.2814 (2)	0.81597 (12)	0.0555 (8)	
O6	0.6840 (2)	0.24327 (14)	0.80900 (10)	0.0333 (6)	
O7	0.3600 (2)	0.31285 (15)	0.49024 (10)	0.0349 (6)	
O8	0.5516 (2)	0.3137 (2)	0.48444 (13)	0.0710 (10)	
N1	0.6832 (3)	0.09923 (18)	0.90061 (15)	0.0374 (7)	
H1N	0.653 (3)	0.100 (2)	0.9325 (13)	0.045*	
N2	0.8942 (3)	0.13883 (19)	0.86124 (13)	0.0343 (7)	
H2N	0.954 (2)	0.153 (2)	0.8863 (15)	0.041*	
N3	0.8662 (3)	0.32447 (18)	0.90502 (14)	0.0353 (7)	
H3N	0.9388 (19)	0.317 (2)	0.9209 (17)	0.042*	
N4	0.6460 (2)	0.28272 (18)	0.93980 (13)	0.0327 (6)	
H4N	0.582 (2)	0.275 (2)	0.9139 (15)	0.039*	

### Atomic displacement parameters (Å^2^)

**Table:**

	*U*^11^	*U*^22^	*U*^33^	*U*^12^	*U*^13^	*U*^23^
Ni1	0.0219 (2)	0.0338 (2)	0.0203 (2)	−0.00261 (19)	0.00421 (14)	0.00109 (18)
C1	0.041 (2)	0.044 (2)	0.038 (2)	−0.0139 (18)	0.0019 (17)	0.0005 (17)
C2	0.071 (3)	0.079 (3)	0.053 (3)	−0.038 (3)	0.025 (2)	−0.024 (2)
C3	0.086 (4)	0.068 (3)	0.074 (4)	−0.034 (3)	0.024 (3)	−0.037 (3)
C4	0.067 (3)	0.050 (3)	0.069 (3)	−0.027 (2)	0.008 (3)	−0.007 (2)
C5	0.052 (3)	0.058 (3)	0.061 (3)	−0.022 (2)	0.011 (2)	0.000 (2)
C6	0.041 (2)	0.048 (2)	0.055 (3)	−0.0132 (19)	0.0094 (19)	−0.007 (2)
C9	0.037 (2)	0.062 (3)	0.060 (3)	−0.013 (2)	0.017 (2)	0.003 (2)
C10	0.058 (3)	0.063 (3)	0.076 (3)	−0.007 (2)	0.037 (3)	−0.001 (3)
C11	0.041 (2)	0.055 (2)	0.032 (2)	−0.0009 (19)	0.0169 (16)	−0.0058 (17)
C12	0.042 (2)	0.092 (4)	0.066 (3)	0.002 (2)	0.028 (2)	−0.016 (3)
C13	0.080 (4)	0.093 (4)	0.105 (5)	0.010 (3)	0.058 (4)	−0.029 (4)
C14	0.098 (5)	0.088 (4)	0.083 (4)	−0.008 (3)	0.051 (4)	−0.038 (3)
C15	0.065 (3)	0.073 (3)	0.062 (3)	−0.015 (3)	0.029 (2)	−0.024 (3)
C16	0.039 (2)	0.054 (2)	0.0284 (18)	−0.0033 (18)	0.0130 (15)	−0.0051 (17)
C17	0.0337 (19)	0.057 (2)	0.0275 (19)	0.0029 (18)	0.0086 (15)	0.0014 (16)
C18	0.060 (3)	0.038 (2)	0.045 (2)	0.003 (2)	0.024 (2)	−0.0016 (18)
C19	0.062 (3)	0.043 (2)	0.042 (2)	−0.013 (2)	0.022 (2)	−0.0072 (18)
C20	0.055 (2)	0.046 (2)	0.034 (2)	−0.008 (2)	0.0196 (18)	0.0013 (17)
C21	0.059 (3)	0.037 (2)	0.037 (2)	−0.0095 (19)	0.0198 (19)	−0.0019 (16)
C22	0.076 (3)	0.041 (2)	0.072 (3)	0.001 (2)	0.036 (3)	0.001 (2)
C23	0.110 (5)	0.034 (2)	0.104 (5)	0.000 (3)	0.054 (4)	−0.002 (3)
C24	0.093 (4)	0.049 (3)	0.095 (4)	−0.024 (3)	0.041 (3)	−0.011 (3)
C25	0.064 (3)	0.056 (3)	0.079 (3)	−0.023 (2)	0.028 (3)	−0.009 (2)
C26	0.060 (3)	0.038 (2)	0.053 (3)	−0.012 (2)	0.022 (2)	0.0032 (18)
C27	0.041 (3)	0.061 (3)	0.103 (4)	−0.012 (2)	0.017 (3)	0.005 (3)
C28	0.041 (3)	0.078 (4)	0.112 (5)	0.004 (3)	−0.007 (3)	−0.016 (3)
C29	0.043 (2)	0.047 (2)	0.044 (2)	0.0136 (19)	0.0114 (18)	0.0012 (18)
C30	0.046 (3)	0.079 (3)	0.064 (3)	0.017 (2)	0.020 (2)	0.008 (3)
C31	0.071 (3)	0.079 (3)	0.059 (3)	0.035 (3)	0.029 (3)	0.000 (3)
C32	0.079 (4)	0.061 (3)	0.068 (3)	0.018 (3)	0.034 (3)	−0.007 (2)
C33	0.065 (3)	0.052 (2)	0.057 (3)	0.007 (2)	0.026 (2)	−0.004 (2)
C34	0.047 (2)	0.046 (2)	0.038 (2)	0.0129 (19)	0.0186 (18)	0.0065 (17)
C35	0.040 (2)	0.045 (2)	0.034 (2)	0.0050 (17)	0.0133 (16)	0.0007 (16)
C36	0.068 (2)	0.0466 (17)	0.085 (3)	0.0102 (17)	0.0316 (19)	0.0148 (17)
C37	0.068 (2)	0.0466 (17)	0.085 (3)	0.0102 (17)	0.0316 (19)	0.0148 (17)
C38	0.045 (2)	0.052 (2)	0.041 (2)	−0.020 (2)	0.0005 (18)	0.0051 (18)
C39	0.0295 (18)	0.042 (2)	0.0235 (17)	0.0001 (16)	0.0031 (14)	0.0026 (14)
C40	0.0281 (16)	0.0335 (17)	0.0256 (16)	0.0029 (16)	0.0026 (13)	−0.0014 (14)
C41	0.0271 (17)	0.057 (2)	0.0259 (18)	0.0063 (17)	0.0065 (14)	−0.0033 (16)
C42	0.0227 (16)	0.053 (2)	0.0271 (18)	0.0100 (16)	−0.0004 (14)	−0.0020 (16)
C43	0.0266 (17)	0.0344 (17)	0.0252 (17)	0.0023 (15)	0.0013 (13)	0.0025 (14)
C44	0.0266 (18)	0.049 (2)	0.0247 (18)	0.0046 (16)	0.0034 (14)	0.0023 (15)
C45	0.0240 (17)	0.059 (2)	0.0280 (18)	−0.0045 (17)	0.0051 (14)	0.0066 (16)
C46	0.0234 (16)	0.051 (2)	0.0304 (18)	−0.0007 (16)	−0.0030 (14)	0.0055 (16)
O1	0.0562 (14)	0.0712 (15)	0.0840 (17)	−0.0255 (12)	0.0396 (12)	−0.0315 (13)
O2	0.0562 (14)	0.0712 (15)	0.0840 (17)	−0.0255 (12)	0.0396 (12)	−0.0315 (13)
O3	0.059 (2)	0.0482 (17)	0.096 (3)	−0.0060 (16)	0.0329 (18)	0.0088 (17)
O4	0.0445 (18)	0.061 (2)	0.099 (3)	0.0058 (16)	−0.0041 (17)	−0.0072 (18)
O5	0.0341 (14)	0.103 (2)	0.0301 (14)	0.0175 (16)	0.0081 (11)	0.0096 (15)
O6	0.0264 (12)	0.0468 (14)	0.0250 (12)	0.0031 (11)	0.0017 (10)	0.0051 (10)
O7	0.0254 (12)	0.0561 (15)	0.0222 (12)	0.0011 (11)	0.0022 (9)	−0.0025 (11)
O8	0.0278 (14)	0.157 (3)	0.0285 (15)	0.0042 (18)	0.0065 (12)	0.0067 (18)
N1	0.0338 (17)	0.0397 (17)	0.0370 (18)	−0.0087 (14)	0.0029 (14)	0.0030 (14)
N2	0.0317 (16)	0.0443 (17)	0.0275 (16)	0.0010 (15)	0.0077 (12)	0.0011 (13)
N3	0.0359 (17)	0.0403 (16)	0.0317 (17)	−0.0076 (15)	0.0119 (13)	−0.0028 (13)
N4	0.0274 (15)	0.0459 (17)	0.0261 (15)	0.0005 (15)	0.0084 (11)	0.0014 (14)

### Geometric parameters (Å, °)

**Table:**

Ni1—O6	2.049 (2)	C24—H24	0.9300
Ni1—O7^i^	2.089 (2)	C25—C26	1.376 (6)
Ni1—N1	2.131 (3)	C25—H25	0.9300
Ni1—N3	2.142 (3)	C26—O3	1.377 (5)
Ni1—N4	2.151 (3)	C27—O3	1.399 (5)
Ni1—N2	2.160 (3)	C27—C28	1.465 (7)
C1—C6	1.377 (6)	C27—H27A	0.9700
C1—C2	1.384 (6)	C27—H27B	0.9700
C1—C38	1.508 (5)	C28—O4	1.359 (5)
C2—C3	1.393 (6)	C28—H28A	0.9700
C2—H2	0.9300	C28—H28B	0.9700
C3—C4	1.356 (7)	C29—O4	1.377 (5)
C3—H3	0.9300	C29—C34	1.382 (6)
C4—C5	1.370 (6)	C29—C30	1.394 (6)
C4—H4	0.9300	C30—C31	1.376 (7)
C5—C6	1.374 (5)	C30—H30	0.9300
C5—H5	0.9300	C31—C32	1.369 (7)
C6—O1	1.385 (5)	C31—H31	0.9300
C9—O1	1.383 (5)	C32—C33	1.383 (6)
C9—C10	1.471 (6)	C32—H32	0.9300
C9—H9A	0.9700	C33—C34	1.402 (6)
C9—H9B	0.9700	C33—H33	0.9300
C10—O2	1.382 (5)	C34—C35	1.506 (5)
C10—H10A	0.9700	C35—N2	1.482 (4)
C10—H10B	0.9700	C35—H35A	0.9700
C11—C16	1.381 (5)	C35—H35B	0.9700
C11—C12	1.383 (6)	C36—C37	1.469 (6)
C11—O2	1.389 (5)	C36—N2	1.500 (5)
C12—C13	1.380 (7)	C36—H36A	0.9700
C12—H12	0.9300	C36—H36B	0.9700
C13—C14	1.366 (8)	C37—N1	1.450 (5)
C13—H13	0.9300	C37—H37A	0.9700
C14—C15	1.380 (7)	C37—H37B	0.9700
C14—H14	0.9300	C38—N1	1.474 (5)
C15—C16	1.389 (6)	C38—H38A	0.9700
C15—H15	0.9300	C38—H38B	0.9700
C16—C17	1.512 (5)	C39—O5	1.236 (4)
C17—N4	1.485 (4)	C39—O6	1.274 (4)
C17—H17A	0.9700	C39—C40	1.519 (4)
C17—H17B	0.9700	C40—C46	1.382 (5)
C18—N4	1.481 (5)	C40—C41	1.386 (4)
C18—C19	1.508 (6)	C41—C42	1.382 (5)
C18—H18A	0.9700	C41—H41	0.9300
C18—H18B	0.9700	C42—C43	1.377 (5)
C19—N3	1.478 (5)	C42—H42	0.9300
C19—H19A	0.9700	C43—C45	1.393 (4)
C19—H19B	0.9700	C43—C44	1.523 (5)
C20—N3	1.479 (5)	C44—O8	1.228 (4)
C20—C21	1.510 (5)	C44—O7	1.267 (4)
C20—H20A	0.9700	C45—C46	1.387 (5)
C20—H20B	0.9700	C45—H45	0.9300
C21—C26	1.384 (6)	C46—H46	0.9300
C21—C22	1.390 (6)	O7—Ni1^ii^	2.089 (2)
C22—C23	1.401 (7)	N1—H1N	0.83 (2)
C22—H22	0.9300	N2—H2N	0.81 (2)
C23—C24	1.343 (7)	N3—H3N	0.84 (2)
C23—H23	0.9300	N4—H4N	0.83 (2)
C24—C25	1.376 (7)		
			
O6—Ni1—O7^i^	177.49 (10)	C28—C27—H27A	109.6
O6—Ni1—N1	95.97 (11)	O3—C27—H27B	109.6
O7^i^—Ni1—N1	86.54 (11)	C28—C27—H27B	109.6
O6—Ni1—N3	89.42 (11)	H27A—C27—H27B	108.1
O7^i^—Ni1—N3	88.08 (11)	O4—C28—C27	111.5 (4)
N1—Ni1—N3	174.59 (12)	O4—C28—H28A	109.3
O6—Ni1—N4	92.13 (10)	C27—C28—H28A	109.3
O7^i^—Ni1—N4	87.37 (10)	O4—C28—H28B	109.3
N1—Ni1—N4	97.09 (12)	C27—C28—H28B	109.3
N3—Ni1—N4	83.23 (12)	H28A—C28—H28B	108.0
O6—Ni1—N2	90.38 (10)	O4—C29—C34	116.1 (3)
O7^i^—Ni1—N2	90.20 (10)	O4—C29—C30	122.3 (4)
N1—Ni1—N2	81.13 (12)	C34—C29—C30	121.5 (4)
N3—Ni1—N2	98.32 (12)	C31—C30—C29	119.0 (5)
N4—Ni1—N2	177.07 (11)	C31—C30—H30	120.5
C6—C1—C2	117.0 (4)	C29—C30—H30	120.5
C6—C1—C38	120.8 (4)	C32—C31—C30	121.2 (4)
C2—C1—C38	122.2 (4)	C32—C31—H31	119.4
C1—C2—C3	121.4 (4)	C30—C31—H31	119.4
C1—C2—H2	119.3	C31—C32—C33	119.3 (5)
C3—C2—H2	119.3	C31—C32—H32	120.4
C4—C3—C2	119.8 (5)	C33—C32—H32	120.4
C4—C3—H3	120.1	C32—C33—C34	121.4 (5)
C2—C3—H3	120.1	C32—C33—H33	119.3
C3—C4—C5	119.9 (4)	C34—C33—H33	119.3
C3—C4—H4	120.1	C29—C34—C33	117.5 (4)
C5—C4—H4	120.1	C29—C34—C35	120.7 (4)
C4—C5—C6	120.1 (5)	C33—C34—C35	121.8 (4)
C4—C5—H5	119.9	N2—C35—C34	115.0 (3)
C6—C5—H5	119.9	N2—C35—H35A	108.5
C5—C6—C1	121.8 (4)	C34—C35—H35A	108.5
C5—C6—O1	123.3 (4)	N2—C35—H35B	108.5
C1—C6—O1	114.9 (3)	C34—C35—H35B	108.5
O1—C9—C10	111.3 (3)	H35A—C35—H35B	107.5
O1—C9—H9A	109.4	C37—C36—N2	115.0 (4)
C10—C9—H9A	109.4	C37—C36—H36A	108.5
O1—C9—H9B	109.4	N2—C36—H36A	108.5
C10—C9—H9B	109.4	C37—C36—H36B	108.5
H9A—C9—H9B	108.0	N2—C36—H36B	108.5
O2—C10—C9	112.6 (4)	H36A—C36—H36B	107.5
O2—C10—H10A	109.1	N1—C37—C36	110.8 (4)
C9—C10—H10A	109.1	N1—C37—H37A	109.5
O2—C10—H10B	109.1	C36—C37—H37A	109.5
C9—C10—H10B	109.1	N1—C37—H37B	109.5
H10A—C10—H10B	107.8	C36—C37—H37B	109.5
C16—C11—C12	121.8 (4)	H37A—C37—H37B	108.1
C16—C11—O2	116.6 (3)	N1—C38—C1	115.0 (3)
C12—C11—O2	121.6 (4)	N1—C38—H38A	108.5
C13—C12—C11	119.2 (5)	C1—C38—H38A	108.5
C13—C12—H12	120.4	N1—C38—H38B	108.5
C11—C12—H12	120.4	C1—C38—H38B	108.5
C14—C13—C12	119.8 (5)	H38A—C38—H38B	107.5
C14—C13—H13	120.1	O5—C39—O6	126.5 (3)
C12—C13—H13	120.1	O5—C39—C40	118.3 (3)
C13—C14—C15	120.8 (5)	O6—C39—C40	115.2 (3)
C13—C14—H14	119.6	C46—C40—C41	118.6 (3)
C15—C14—H14	119.6	C46—C40—C39	122.0 (3)
C14—C15—C16	120.5 (5)	C41—C40—C39	119.3 (3)
C14—C15—H15	119.8	C42—C41—C40	120.5 (3)
C16—C15—H15	119.8	C42—C41—H41	119.8
C11—C16—C15	117.8 (4)	C40—C41—H41	119.8
C11—C16—C17	120.7 (3)	C43—C42—C41	121.2 (3)
C15—C16—C17	121.5 (4)	C43—C42—H42	119.4
N4—C17—C16	115.2 (3)	C41—C42—H42	119.4
N4—C17—H17A	108.5	C42—C43—C45	118.5 (3)
C16—C17—H17A	108.5	C42—C43—C44	120.3 (3)
N4—C17—H17B	108.5	C45—C43—C44	121.1 (3)
C16—C17—H17B	108.5	O8—C44—O7	125.2 (3)
H17A—C17—H17B	107.5	O8—C44—C43	119.8 (3)
N4—C18—C19	109.1 (3)	O7—C44—C43	114.9 (3)
N4—C18—H18A	109.9	C46—C45—C43	120.3 (3)
C19—C18—H18A	109.9	C46—C45—H45	119.9
N4—C18—H18B	109.9	C43—C45—H45	119.9
C19—C18—H18B	109.9	C40—C46—C45	120.9 (3)
H18A—C18—H18B	108.3	C40—C46—H46	119.6
N3—C19—C18	108.5 (3)	C45—C46—H46	119.6
N3—C19—H19A	110.0	C9—O1—C6	119.8 (3)
C18—C19—H19A	110.0	C10—O2—C11	116.4 (3)
N3—C19—H19B	110.0	C26—O3—C27	118.1 (3)
C18—C19—H19B	110.0	C28—O4—C29	118.8 (4)
H19A—C19—H19B	108.4	C39—O6—Ni1	132.4 (2)
N3—C20—C21	114.5 (3)	C44—O7—Ni1^ii^	130.8 (2)
N3—C20—H20A	108.6	C37—N1—C38	116.6 (4)
C21—C20—H20A	108.6	C37—N1—Ni1	105.4 (3)
N3—C20—H20B	108.6	C38—N1—Ni1	115.7 (2)
C21—C20—H20B	108.6	C37—N1—H1N	106 (3)
H20A—C20—H20B	107.6	C38—N1—H1N	106 (3)
C26—C21—C22	118.3 (4)	Ni1—N1—H1N	106 (3)
C26—C21—C20	119.8 (4)	C35—N2—C36	110.4 (3)
C22—C21—C20	121.8 (4)	C35—N2—Ni1	118.8 (2)
C21—C22—C23	119.8 (5)	C36—N2—Ni1	107.4 (2)
C21—C22—H22	120.1	C35—N2—H2N	112 (3)
C23—C22—H22	120.1	C36—N2—H2N	112 (3)
C24—C23—C22	120.2 (5)	Ni1—N2—H2N	96 (3)
C24—C23—H23	119.9	C19—N3—C20	112.3 (3)
C22—C23—H23	119.9	C19—N3—Ni1	104.5 (2)
C23—C24—C25	121.1 (5)	C20—N3—Ni1	119.3 (2)
C23—C24—H24	119.5	C19—N3—H3N	106 (3)
C25—C24—H24	119.5	C20—N3—H3N	104 (3)
C26—C25—C24	119.2 (5)	Ni1—N3—H3N	110 (3)
C26—C25—H25	120.4	C18—N4—C17	112.2 (3)
C24—C25—H25	120.4	C18—N4—Ni1	105.4 (2)
C25—C26—O3	123.3 (4)	C17—N4—Ni1	118.3 (2)
C25—C26—C21	121.3 (4)	C18—N4—H4N	109 (3)
O3—C26—C21	115.4 (3)	C17—N4—H4N	110 (3)
O3—C27—C28	110.2 (4)	Ni1—N4—H4N	101 (3)
O3—C27—H27A	109.6		
			
C6—C1—C2—C3	1.5 (7)	C41—C40—C46—C45	−2.4 (5)
C38—C1—C2—C3	−177.8 (4)	C39—C40—C46—C45	174.2 (3)
C1—C2—C3—C4	0.3 (8)	C43—C45—C46—C40	−0.8 (6)
C2—C3—C4—C5	−0.9 (8)	C10—C9—O1—C6	−178.7 (4)
C3—C4—C5—C6	−0.4 (8)	C5—C6—O1—C9	−34.0 (6)
C4—C5—C6—C1	2.3 (7)	C1—C6—O1—C9	149.7 (4)
C4—C5—C6—O1	−173.8 (4)	C9—C10—O2—C11	178.9 (4)
C2—C1—C6—C5	−2.8 (6)	C16—C11—O2—C10	131.3 (4)
C38—C1—C6—C5	176.5 (4)	C12—C11—O2—C10	−50.8 (6)
C2—C1—C6—O1	173.6 (4)	C25—C26—O3—C27	−36.7 (7)
C38—C1—C6—O1	−7.0 (6)	C21—C26—O3—C27	143.7 (4)
O1—C9—C10—O2	41.1 (6)	C28—C27—O3—C26	−160.0 (4)
C16—C11—C12—C13	−1.6 (7)	C27—C28—O4—C29	176.2 (4)
O2—C11—C12—C13	−179.3 (5)	C34—C29—O4—C28	140.7 (5)
C11—C12—C13—C14	−0.5 (9)	C30—C29—O4—C28	−41.9 (7)
C12—C13—C14—C15	1.7 (10)	O5—C39—O6—Ni1	−4.2 (6)
C13—C14—C15—C16	−0.9 (9)	C40—C39—O6—Ni1	175.8 (2)
C12—C11—C16—C15	2.4 (6)	N1—Ni1—O6—C39	78.3 (3)
O2—C11—C16—C15	−179.8 (4)	N3—Ni1—O6—C39	−102.3 (3)
C12—C11—C16—C17	−178.1 (4)	N4—Ni1—O6—C39	−19.1 (3)
O2—C11—C16—C17	−0.2 (6)	N2—Ni1—O6—C39	159.4 (3)
C14—C15—C16—C11	−1.1 (7)	O8—C44—O7—Ni1^ii^	14.3 (6)
C14—C15—C16—C17	179.3 (5)	C43—C44—O7—Ni1^ii^	−163.6 (2)
C11—C16—C17—N4	83.3 (5)	C36—C37—N1—C38	81.0 (5)
C15—C16—C17—N4	−97.2 (5)	C36—C37—N1—Ni1	−48.9 (5)
N4—C18—C19—N3	61.8 (4)	C1—C38—N1—C37	54.7 (5)
N3—C20—C21—C26	71.6 (5)	C1—C38—N1—Ni1	179.6 (3)
N3—C20—C21—C22	−110.4 (4)	O6—Ni1—N1—C37	122.4 (3)
C26—C21—C22—C23	−0.6 (7)	O7^i^—Ni1—N1—C37	−57.8 (3)
C20—C21—C22—C23	−178.6 (4)	N4—Ni1—N1—C37	−144.7 (3)
C21—C22—C23—C24	0.8 (8)	N2—Ni1—N1—C37	32.9 (3)
C22—C23—C24—C25	−0.9 (9)	O6—Ni1—N1—C38	−8.1 (3)
C23—C24—C25—C26	0.8 (8)	O7^i^—Ni1—N1—C38	171.7 (3)
C24—C25—C26—O3	179.8 (5)	N4—Ni1—N1—C38	84.8 (3)
C24—C25—C26—C21	−0.6 (7)	N2—Ni1—N1—C38	−97.6 (3)
C22—C21—C26—C25	0.5 (7)	C34—C35—N2—C36	−66.6 (4)
C20—C21—C26—C25	178.6 (4)	C34—C35—N2—Ni1	168.8 (3)
C22—C21—C26—O3	−179.9 (4)	C37—C36—N2—C35	−142.5 (4)
C20—C21—C26—O3	−1.8 (6)	C37—C36—N2—Ni1	−11.6 (5)
O3—C27—C28—O4	56.8 (7)	O6—Ni1—N2—C35	18.2 (3)
O4—C29—C30—C31	−177.0 (4)	O7^i^—Ni1—N2—C35	−159.3 (3)
C34—C29—C30—C31	0.2 (7)	N1—Ni1—N2—C35	114.2 (3)
C29—C30—C31—C32	1.8 (7)	N3—Ni1—N2—C35	−71.2 (3)
C30—C31—C32—C33	−1.4 (8)	O6—Ni1—N2—C36	−107.9 (3)
C31—C32—C33—C34	−1.1 (7)	O7^i^—Ni1—N2—C36	74.6 (3)
O4—C29—C34—C33	174.9 (4)	N1—Ni1—N2—C36	−11.9 (3)
C30—C29—C34—C33	−2.4 (6)	N3—Ni1—N2—C36	162.6 (3)
O4—C29—C34—C35	−4.8 (6)	C18—C19—N3—C20	83.7 (4)
C30—C29—C34—C35	177.8 (4)	C18—C19—N3—Ni1	−47.0 (3)
C32—C33—C34—C29	2.9 (6)	C21—C20—N3—C19	61.7 (4)
C32—C33—C34—C35	−177.4 (4)	C21—C20—N3—Ni1	−175.6 (3)
C29—C34—C35—N2	−91.4 (4)	O6—Ni1—N3—C19	111.2 (2)
C33—C34—C35—N2	88.8 (5)	O7^i^—Ni1—N3—C19	−68.6 (2)
N2—C36—C37—N1	41.6 (6)	N4—Ni1—N3—C19	19.0 (2)
C6—C1—C38—N1	76.5 (5)	N2—Ni1—N3—C19	−158.5 (2)
C2—C1—C38—N1	−104.2 (5)	O6—Ni1—N3—C20	−15.3 (3)
O5—C39—C40—C46	166.5 (4)	O7^i^—Ni1—N3—C20	164.9 (3)
O6—C39—C40—C46	−13.6 (5)	N4—Ni1—N3—C20	−107.5 (3)
O5—C39—C40—C41	−16.9 (5)	N2—Ni1—N3—C20	75.0 (3)
O6—C39—C40—C41	163.0 (3)	C19—C18—N4—C17	89.0 (4)
C46—C40—C41—C42	3.2 (5)	C19—C18—N4—Ni1	−41.1 (3)
C39—C40—C41—C42	−173.5 (3)	C16—C17—N4—C18	69.4 (4)
C40—C41—C42—C43	−0.8 (6)	C16—C17—N4—Ni1	−167.5 (2)
C41—C42—C43—C45	−2.5 (5)	O6—Ni1—N4—C18	−77.3 (2)
C41—C42—C43—C44	172.8 (3)	O7^i^—Ni1—N4—C18	100.2 (2)
C42—C43—C44—O8	−177.9 (4)	N1—Ni1—N4—C18	−173.6 (2)
C45—C43—C44—O8	−2.8 (6)	N3—Ni1—N4—C18	11.9 (2)
C42—C43—C44—O7	0.2 (5)	O6—Ni1—N4—C17	156.3 (2)
C45—C43—C44—O7	175.3 (3)	O7^i^—Ni1—N4—C17	−26.1 (2)
C42—C43—C45—C46	3.2 (5)	N1—Ni1—N4—C17	60.0 (3)
C44—C43—C45—C46	−172.0 (3)	N3—Ni1—N4—C17	−114.5 (3)

Symmetry codes: (i) *x*+1/2, −*y*+1/2, *z*+1/2; (ii) *x*−1/2, −*y*+1/2, *z*−1/2.

### Hydrogen-bond geometry (Å, °)

**Table:**

*D*—H···*A*	*D*—H	H···*A*	*D*···*A*	*D*—H···*A*
N1—H1N···O1	0.83 (2)	2.58 (4)	3.101 (4)	122 (3)
N2—H2N···O8^i^	0.81 (2)	2.26 (2)	3.003 (4)	152 (4)
N3—H3N···O3	0.84 (2)	2.47 (3)	3.044 (4)	127 (3)
N4—H4N···O5	0.83 (2)	2.14 (3)	2.862 (4)	144 (4)
C10—H10A···O8^iii^	0.97	2.40	3.359 (6)	172
C12—H12···O8^iii^	0.93	2.60	3.508 (6)	166
C28—H28B···O2^iv^	0.97	2.47	3.210 (7)	133
C28—H28A···O5^iv^	0.97	2.47	3.342 (6)	149
C35—H35A···O4	0.97	2.38	2.761 (5)	103
C37—H37B···O7^i^	0.97	2.47	2.985 (6)	113
C42—H42···O7	0.93	2.44	2.752 (4)	100

Symmetry codes: (i) *x*+1/2, −*y*+1/2, *z*+1/2; (iii) *x*−1/2, −*y*+1/2, *z*+1/2; (iv) *x*+1, *y*, *z*.
